# Automated VSS-based Burn Scar Assessment using Combined Texture and Color Features of Digital Images in Error-Correcting Output Coding

**DOI:** 10.1038/s41598-017-16914-0

**Published:** 2017-12-01

**Authors:** Tuan D. Pham, Matilda Karlsson, Caroline M. Andersson, Robin Mirdell, Folke Sjoberg

**Affiliations:** 1 Linkoping University, Department of Biomedical Engineering, 58283 Linkoping, Sweden; 2 Linkoping University Hospital, Linkoping Burn Centre, 58185 Linkoping, Sweden; 3 Linkoping University, Department of Plastic Surgery, Hand Surgery, and Burns, 58185 Linkoping, Sweden; 4 Linkoping University, Department of Clinical and Experimental Medicine, 58185 Linkoping, Sweden

## Abstract

Assessment of burn scars is an important study in both medical research and clinical settings because it can help determine response to burn treatment and plan optimal surgical procedures. Scar rating has been performed using both subjective observations and objective measuring devices. However, there is still a lack of consensus with respect to the accuracy, reproducibility, and feasibility of the current methods. Computerized scar assessment appears to have potential for meeting such requirements but has been rarely found in literature. In this paper an image analysis and pattern classification approach for automating burn scar rating based on the Vancouver Scar Scale (VSS) was developed. Using the image data of pediatric patients, a rating accuracy of 85% was obtained, while 92% and 98% were achieved for the tolerances of one VSS score and two VSS scores, respectively. The experimental results suggest that the proposed approach is very promising as a tool for clinical burn scar assessment that is reproducible and cost-effective.

## Introduction

Burns are known to be one of the most common domestic injuries, especially among children, and a stressful problem of critical care^1^. Burns are characterized by skin damage that terminates skin cells of the affected area. The classification of burns includes 4 categories^2^: epidermal, superficial partial-thickness, deep partial-thickness, and full-thickness. The recovery from a burn injury depends on the level of severity, where more severe burns require the emergency of medical treatment in order to avoid complications and death. After burn healing, scarring is a concerning issue of both body image and physical function to patients with burn injuries. In addition, the quantification of the characteristics of burn scars has important implications for monitoring of the healing process, comparison and assessment of different surgical interventions that would turn out to enable more optimal treatment and provide more effective pre-operative counseling. Thus, it is important to evaluate the severity of burn scars, and several tools and instruments have been developed for assessing one or more aspects of burn scars to improve the quality of life of the patient^3^.

For burn scar treatment, it is necessary to make accurate and reproducible clinical findings of scar assessment so that various interventions and treatments can be consistently interpreted and compared on a universal basis^4^. Since the introduction of the Vancouver Scar Scale (VSS)^5^, more than ten scar rating scales have been developed to contribute to the standardization of scar therapy and enhancement of the assessment^4^. A review of scar scales and scar measurement instruments suggested that there is a need for the development of an optimal scar scoring system so that pathologic scarring can be better treated^6^. In addition to the subjective assessment of burn scars, objective scar evaluations have been carried out by measuring the physical properties of the scar such as its height or its vascularity. In general, measures of scar severity are suggested to be based on color (pigmentation), dimensions (area, thickness and volume), texture, biomechanical properties (pliability and elasticity), pathophysiological disturbances (oxygen tension, water loss and moisture content), tissue microstructure, and pain^7^. However, it is still difficult to obtain an overall scar rating using objective measurement devices because many expensive instruments such as the tonometer, dermaspectrometer, or chromometer are required, in addition to the requirement of experienced users, making the assessment time-consuming and impractical in busy clinical settings^8–10^.

There are few attempts in developing computerized image analysis of wound healing and wound assessment^11–14^, and even less in computerized quantification of burn scar assessment since the work on a finite element modeling of scar image and elasticity for determining a relative elastic index that shows some correlation with the VSS between 1 and 5 scored by a physician over four patients^15^. Being motivated by the demand for developing effective methods for burn scar assessment, we present here some methods for extracting texture and color features of VSS-rated burn scars in digital images, which can be learned by computer algorithms for automated scar rating. It appears that the proposed method is the first of its type in an effort to develop a machine-learning assisted tool for the VSS-based rating of scar characteristics. Although the VSS is one of the most commonly adopted methods for burn scar assessment in clinical practice to date^4,7,16^, it has drawbacks. Several studies have shown that the VSS does not have strong evidence for validity and reliability, in particular reference to large or irregular scars^8,17^, and it does not take into account other information such as pain and itch, and other functional and psychological conditions of scars^4^. It should be therefore pointed out that this study did not attempt to improve the reliability of the VSS, but to automate the VSS-based burn scar assessment by machine learning of the knowledge given by clinical experts, where the computerized assessment can be reproducible and economical.

## Methods

### Ethics

This study was approved by the Regional Ethical Board (REB), and conducted in compliance with the “Ethical principles for medical research involving human subjects” of the Helsinki Declaration. Guardians for research subjects for this study were provided a consent form describing this study and providing sufficient information for subjects to make an informed decision about their child’s participation in this study. The consent form was approved by the REB for the study. Before a subject underwent any study procedure, an informed consent discussion was conducted and written informed consent was obtained from the legal guardians attending at the visit.

### Participants’ characteristics

The participants were hospitalized or outward pediatric burn patients with dermal or full thickness thermal burns who met the following entrance criteria.

#### Inclusion criteria

1) Males or females of 6 months-6 years old, 2) thermal burns caused by scalding, 3) partial thickness (superficial or deep dermal) or full thickness burn wounds requiring temporary skin cover according to the burn surgeon responsible for the patient, 4) signed informed consent from all legal guardians, and 5) burn of no more than 72 hours old after injury.

#### Exclusion criteria

1) Other severe cutaneous trauma at the same site as the burn (to be treated) or previous burn at the same treatment site, 2) inappropriate to participate in the study, for any reason, in the opinion of the investigator, 3) severe cognitive dysfunction or psychiatric disorder, 4) a skin disorder that is chronic or currently active and which the investigator considers will adversely affect the healing of the wound or involves the areas to be examined in this trial, and 5) patients with a known sensitivity to silver.

### Subjective scar assessment

The scars of 6 months after injury were subjectively evaluated using the Vancouver Scar Scale (VSS)^5^, of which description is shown in Table 1. The VSS was designed based on physical parameters relating to the wound healing and maturation, cosmetic appearance of wounds, and the function of the healed skin^5^. The VSS characterizes burn scars by their pigmentation, vascularity, pliability, and height. For the measure of pliability, which is one of the biomechanical properties of the scar, several measurement techniques can be used such as suction (the cutometer was used in this study), tonometry, torsion, adherence, reviscometry, ballistometry, quantitative electrical methods, as well as ultrasound and magnetic resonance imaging^7,18^. The resemblance to normal skin has the score of 0, while a greater score indicates a greater pathologic condition of the burn scar. These labeled scars were used for the machine training and validation of the automated scar assessment in this study.

**Table 1:** The Vancouver Scar Scale (VSS)^5^.

Scar characteristics		Score
Pigmentation	Normal color (resembling nearby skin)	0
	Hypopigmentation	1
	Hyperpigmentation	2
Vascularity	Normal	0
	Pink	1
	Red	2
	Purple	3
Pliability	Normal	0
	Supple	1
	Yielding	2
	Firm	3
	Banding	4
	Contracture	5
Height	Normal (flat)	0
	<2 mm	1
	2 mm–5 mm	2
	>5 mm	3
Maximum total score		13

### Burn scar images

Each wound site was photographed using a digital camera COOLPIX P500 (Nikon Corporation, 2011) with flash card memory. The photographs were taken using the zoom facility, where the camera was as near as possible to the patient while including the entire treated area. The time the patients had been sitting between undressing to the point of evaluation and the time the photos were taken was standardized to be 10 minutes to maintain the consistency of the VSS-based assessment according to the scar color appearance.

The burn scars of 13 patients were selected in this study based on their available image data. The areas of the scars include abdomen, palmar right arm wrist, elbow pit, chest, shoulder, neck, back, chest/flank, lower arm, upper arm, axilla, and buttock. The regions of the scars were manually cropped out of the original images of the partial bodies. The numbers of the cropped images assessed with the VSS total score of 0 = 3, VSS total score of 1 = 9, VSS total score of 2 = 2, VSS total score of 4 = 1, VSS total score of 5 = 1, VSS total score of 7 = 5, VSS total score of 8 = 1, and VSS total score of 9 = 2.

**Figure 1:**
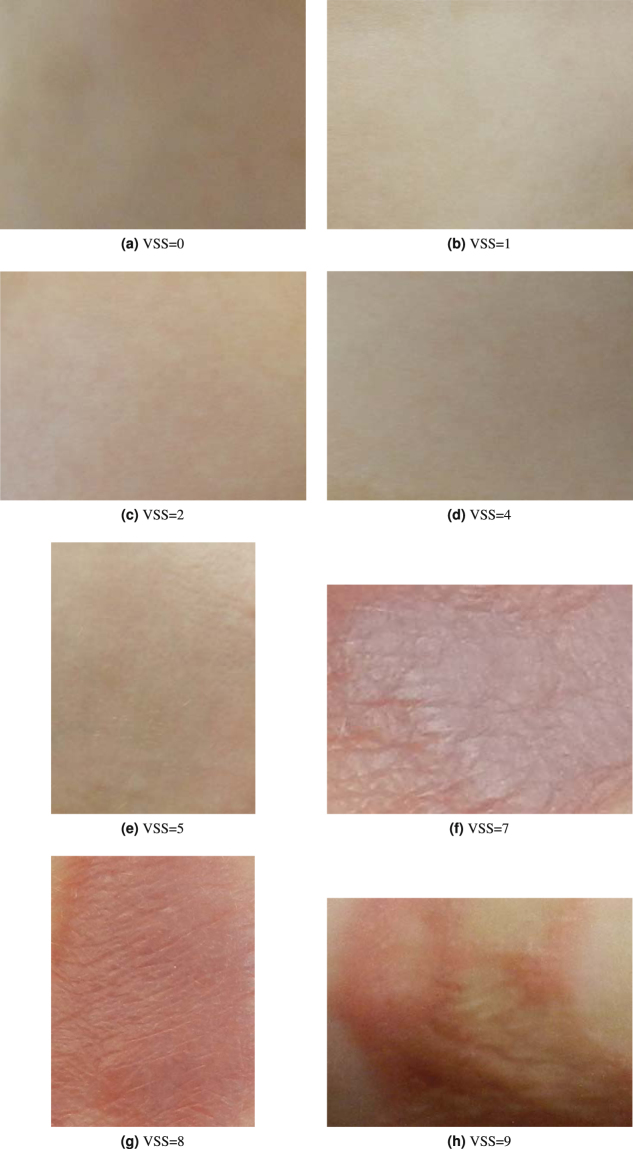
Digital images of 8 VSS classes of burn scars.

To increase the sample size for machine learning and validation, each cropped image was divided into 9 sub-images of equal size, giving the total of 24 × 9 = 216 sub-images. This division of images has been a practice adopted for classifying images with limited samples^19,20^. Figure 1 shows examples of the cropped images of the burn scars of 8 different VSS total scores.

### Texture features

#### Gray-level co-occurrence matrix

The gray-level co-occurrence matrix (GLCM)^21^ is a commonly used method for texture analysis. The GLCM tries to capture the numbers of pairs of pixels that are separated by a certain distance and direction, and have the same gray levels. The value of a GLCM element is defined as

1 $${c}_{h}(p,q)=\sum _{(u,v)|{h}_{uv}=h}^{n(h)}({f}_{u}=p)\wedge ({f}_{v}=q),{\rm{\forall }}p,q\in {\mathscr{L}},$$

in which *f*_*u*_ and *f*_*v*_ are pixels at locations *u* and *v* and having intensity values *p* and *q*, respectively, which are separated by the lag *h*, ∧ stands for the logical AND operator, ${\mathcal L}$ is the set of the image intensity levels, and *n*(*h*) is the total number of pairs of pixels offset by *h*.

The probability of the co-occurrence of *p* and *q* with respect to *h* is

2 $${p}_{h}(p,q)=\frac{{c}_{h}(p,q)}{n(h)}.$$

where *n*(*h*) is the total number of pairs of pixels offset by *h*.

The probabilities of the GLCM defined in Equation (2) allows a variety of definitions of GLCM features. In this study, the following 19 GLCM features were utilized: entropy^21^, energy^21^, correlation^21^, contrast^21^, sum of squares (variance)^21^, sum average^21^, sum variance^21^, sum entropy^21^, difference variance^21^, difference entropy^21^, information measures of correlation^21^, autocorrelation^22^, dissimilarity^22^, homogeneity^22^, cluster prominence^22^, cluster shade^22^, maximum probability^22^, inverse difference^23^, and inverse difference moment normalized^23^.

#### Semi-variogram

The semi-variogram (SV) is a statistic developed in geostatistics^24^. The SV measures the average decreasing similarity between two random variables with increasing distance that separates the two random variables. In terms of probability, the estimation of the SV does not require the knowledge of the mean of the random function. The SV of an image is defined as^20^

3 $$\gamma (h)=\frac{1}{2m(h)}\sum _{i\mathrm{=1}}^{m(h)}{[f({x}_{i})-f({x}_{i}+h)]}^{2},$$

where *f*(*x*_*i*_) is the image intensity at *x*_*i*_, *h* is a distance, and *m*(*h*) is the total number of pairs of pixels separated by *h*.

#### Local binary patterns

The method of local binary patterns (LBP) provides a procedure for quantifying the local image structure, of which local neighborhoods can be encoded with a binarizing process. In general, the value of the LPB code of a pixel at location *u* is defined as^25^

4 $$LPB{(u)}_{V,R}=\sum _{v\mathrm{=0}}^{V-1}b({f}_{v}-{f}_{u}{\mathrm{)2}}^{v},$$

where *f*_*u*_ and *f*_*v*_ are respectively intensity values of the pixel at *u*, and surrounding pixels $v=\mathrm{0,}\ldots ,V-1$, whose center is at *u*, in the circle of radius *R*, and *b*(*x*) is either 1 or 0 if *x* ≥ 0 or otherwise, respectively.

### Color features

#### RGB space

An RGB image is an image of color pixels, where each color pixel is a triplet corresponding to the red, green, and blue components of an RGB image. The RGB color space has been utilized as a color model for computerized analysis of digital photographs of burn scars^7^. The RGB information of the burn scars can be extracted with the first four central moments (mean, variance, skewness, and kurtosis) of the images.

#### HSV space

The RGB color space can be transformed into the HSV (hue, saturation, value) color space, which is considered to be close to human experience and description of color sensation reflecting tint tin (hue), shade (saturation), and tone (value)^26^. As it is a transformation from the RGB space in this study, further color information from the HSV space can be extracted with the use of the histogram.

#### L*a*b space

CIE L*a*b* (CIELAB), which is different from the Lab color space, is a chromatic value color space specified by the International Commission on Illumination (CIE in French), where typically L* = 0 or 100 respectively indicates black or diffuse white, a* has negative or positive value respectively indicates green or magenta, and b* has negative or positive value respectively indicates blue or yellow. CIELAB space describes all the colors visible to the human eye and was used for studying scar measurement techniques in^27^. Similarly, CIELAB is transformed from the RGB space, further color information from the CIELAB space can be extracted with the use of the histogram. For short notation, CIELAB will be denoted as Lab from now on.

### Error-correcting output codes

The approach of error-correcting output codes (ECOC)^28,29^ is an ensemble technique designed for handling multi-class classification problem with binary classifiers. ECOC reduces the classification of multiple classes to a set of binary classifiers by learning one classifier for each pair of classes. In comparison with other multi-class classification models, it was reported that ECOC resulted in better classification accuracy^30^.

ECOC classification requires a coding design and a coding scheme. The coding design determines the classes to be trained with binary learners, and a decoding scheme determines how the results obtained from the binary classifiers are combined by using a loss function. The learners used in this study are support vector machines (SVM), optimized support vector machines (OSVM) using Bayesian optimization^31^, *k*-nearest neighbor (*k*-NN), linear discriminant analysis (LDA), and naive Bayes (NB) methods^32,33^. The classification by the ECOC works by assigning a new observation to the class that minimizes the overall loss for all binary learners.

## Results

Texture and color features of 216 divided images of the burn scars were extracted for machine learning and validation of the automated scar rating. For the texture analysis, the color images were converted to grayscale images. For the GLCM-based texture, 19 features that have been described before were computed for each image, where the gray-level co-occurrence matrices were calculated using an offset of one pixel away to the right of the pixel of interest. The SV for each image was computed with 10 lags in vertical and horizontal directions. Parameters of the LBP were specified as follows: number of neighbors used to compute the LBP for each pixel = 8, radius of circular pattern = 1, no rotation information, and linear interpolation. For the extraction of the color information of the scars, the first four central moments (mean, variance, skewness, and kurtosis) were computed for the RGB space, and histograms with 4 bins computed for the HSV and Lab spaces. All feature vectors were then standardized to have the mean of zero and the standard deviation of one, while still keeping the shape properties (skewness and kurtosis) of the original feature vectors. One-versus-one coding was used in the ECOC method provided in the R2017 Matlab Statistical and Machine Learning toolbox. For the *k*-NN learner, *k* was chosen to be 1. Results and comparisons of different combinations of features classified with various ECOC-learners are presented as follows.

Tables 2 and 3 show the leave-one-out (LOO) cross-validation results of the five ECOC-learners (*k*-NN, LDA, NB, SVM, and OSVM) using texture and color features, respectively. The LDA and NB learners were excluded in further experiments because of their relatively low performance.

**Table 2:** LOO cross-validation of ECOC models using texture features.

Learner	GLCM	LBP	SV	GLCM + SV	GLCM + LBP	LBP + SV	LBP + SV + GLCM
*k*-NN	41.09	73.61	56.02	63.86	81.68	74.07	79.21
LDA	36.14	53.24	54.63	63.37	58.91	57.41	59.41
NB	30.20	53.24	41.20	51.85	69.44	69.91	70.83
SVM	42.57	73.61	49.54	60.19	71.30	74.54	75.46
OSVM	41.58	70.83	58.80	62.04	70.83	79.63	75.46

**Table 3:** LOO cross-validation of ECOC models using color features.

Learner	RGB	HSV	Lab	RGB + HSV	RGB + Lab	RGB + HSV + Lab
*k*-NN	65.28	36.57	31.02	65.28	68.52	68.98
LDA	54.17	40.74	37.04	64.35	57.87	63.89
NB	37.96	30.10	12.50	37.96	18.06	24.07
SVM	58.80	41.20	37.50	63.43	58.33	61.57
OSVM	62.96	43.52	39.35	65.74	64.81	72.69

Tables 4, 5, and 6 show the leave-one-out (LOO) cross-validation results of the ECOC-OSVM, ECOC-SVM, and ECOC-*k*-NN using combinations of texture and color features, respectively.

**Table 4:** LOO cross-validation of ECOC-OSVM using combined texture and color features.

Features	Exact	One-score tolerance	Two-score tolerance
(LBP + SV) + (RGB + HSV + Lab)	**85**.**19**	**92**.**13**	**98**.**15**
(LBP + GLCM) + (RGB + HSV + Lab)	76.85	89.35	96.30
(LBP + SV + GLCM) + (RGB + HSV + Lab)	82.41	91.67	98.15

**Table 5:** LOO cross-validation of ECOC-SVM using combined texture and color features.

Features	Exact	One-score tolerance	Two-score tolerance
(LBP + SV) + (RGB + HSV + Lab)	82.41	90.74	97.69
(LBP + GLCM) + (RGB + HSV + Lab)	74.54	85.65	95.37
(LBP + SV + GLCM) + (RGB + HSV + Lab)	82.87	91.20	97.69

**Table 6:** LOO cross-validation of ECOC-*k*-NN using combined texture and color features.

Features	Exact	One-score tolerance	Two-score tolerance
(LBP + SV) + (RGB + HSV + Lab)	79.17	91.20	95.37
(LBP + GLCM) + (RGB + HSV + Lab)	78.22	87.96	92.59
(LBP + SV + GLCM) + (RGB + HSV + Lab)	81.19	88.43	94.91

Table 7 shows the confusion matrix of the LOO cross-validation of ECOC-OSVM using combined (LBP, SV) and (RGB, HSV, Lab) features, where the accuracy for exact classification = 85.19%, for one-score tolerance = 92.13%, and for two-score tolerance = 98.15%, as shown in Table 4.

**Table 7:** Confusion matrix of LOO cross-validation of ECOC-OSVM using combined (LBP, SV) and (RGB, HSV, Lab) features.

VSS total score	0	1	2	4	5	7	8	9
0	20	5	1	0	1	0	0	0
1	4	74	1	0	2	0	0	0
2	0	1	17	0	0	0	0	0
4	0	0	1	8	0	0	0	0
5	0	0	0	0	9	0	0	0
7	0	1	0	0	0	36	2	6
8	0	0	0	0	0	1	8	0
9	0	0	0	0	0	6	0	12

Table 8 shows the confusion matrix of the LOO cross-validation of ECOC-SVM using combined (LBP, SV, GLCM) and (RGB, HSV, Lab) features, where the accuracy for exact classification = 82.87%, for one-score tolerance = 91.20%, and for two-score tolerance = 97.69%, as shown in Table 5.

**Table 8:** Confusion matrix of LOO cross-validation of ECOC-SVM using combined (LBP, SV, GLCM) and (RGB, HSV, Lab) features.

VSS total score	0	1	2	4	5	7	8	9
0	22	3	1	0	1	0	0	0
1	8	72	1	0	0	0	0	0
2	3	0	15	0	0	0	0	0
4	0	0	0	9	0	0	0	0
5	0	1	0	0	8	0	0	0
7	2	0	0	0	0	35	3	5
8	0	0	0	0	0	3	6	0
9	0	1	0	0	0	5	0	12

Table 9 shows the confusion matrix of the LOO cross-validation of ECOC-*k*-NN using combined (LBP, SV, GLCM) and (RGB, HSV, Lab) features, where the accuracy for exact classification = 81.19%, for one-score tolerance = 88.43%, and for two-score tolerance = 94.91%, as shown in Table 6.

**Table 9:** Confusion matrix of LOO cross-validation of ECOC-*k*-NN using combined (LBP, SV, GLCM) and (RGB, HSV, Lab) features.

VSS total score	0	1	2	4	5	7	8	9
0	18	5	2	1	1	0	0	0
1	3	75	1	2	0	0	0	0
2	0	5	13	0	0	0	0	0
4	0	0	0	9	0	0	0	0
5	2	0	0	0	7	0	0	0
7	2	4	0	0	1	27	6	5
8	0	0	0	0	0	0	9	0
9	0	1	0	0	0	5	0	12

## Discussion

Using textures (Table 2), either GLCM, LBP, or SV, SVM achieved the best result for GLCM, both *k*-NN and SVM achieved the best for LBP, and OSVM resulted in the highest accuracy for SV. The performance of the SVM and NB learners are highest with the combination of the three texture features, OSVM is highest with the combination of LBP and SV, LDA is highest with the combination of GLCM and SV, and *k*-NN has the highest performance with GLCM and LBP. Using color features (Table 3), both *k*-NN and OSVM achieved highest results with the combination of the three color spaces, SVM and LDA are highest with the combination RGB and HSV, and NB is highest with either RGB or the combination of RGB and HSV. Analysis of these experimental results generally suggests the effectiveness in combining individual texture and color features for classifying the images of burn scars.

In the combination of texture and color features for automated assessment of VSS-based burn scars, the OSVM achieved the highest classification rates in terms of the best results of exact assignment = 85.19%, assignment with one-score tolerance = 92.13%, and assignment with two-score tolerance = 98.15%, as shown in Table 4 and the corresponding confusion matrix shown in Table 7. The SVM achieved the second highest classification results, where the best of exact = 82.87%, one-score tolerance = 91.20%, and assignment with two-score tolerance = 97.69%, as shown in Table 5 and the corresponding confusion matrix shown in Table 8. The *k*-NN came third in performance, where the best of exact classification = 81.19%, one-score tolerance = 88.43%, and assignment with two-score tolerance = 94.91%, as shown in Table 6 and the corresponding confusion matrix shown in Table 9. The best results obtained from the OVSM, SVM, and *k*-NN are based on the combinations of (LBP, SV, RGB, HSV, and Lab), (LBP, SV, GLCM, RGB, HSV, and Lab), and (LBP, SV, GLCM, RGB, HSV, and Lab), respectively. The results reflect the OSVM is most favorable in using the complimentary LBP and SV texture features for the classification of burn scar images, as by mathematical definitions, this can be explained in that the SV statistic expresses the global spatial structure of an image, while the LBP method attempts to gain insight into the local image structure.

As shown in the three confusion matrices (Tables 7, 8, and 9), the classification results are distributed along the main diagonals of the matrices, particularly for the OSVM (Table 7), where only small numbers of misclassified samples are far off the main diagonal. Such distributions of the classified samples indicate the strong performance of the classifiers even in case of misclassification.

The above expression is based on the assumption that the spatial autocorrelation structure is isotropic, which implies the semi-variogram depends only on the magnitude of the lag (*h*). When the spatial autocorrelation pattern changes in different directions in the sampling space, an anisotropic semi-variogram can be of more appropriate use. Geometric and zonal properties of anisotropy have been introduced to model the semivariogram^34^. The geometric anisotropy is imposed when the range of the semivariogram varies in different directions. The zonal anisotropy is a phenomenon when the range and sill of the semivariogram are not constant. Furthermore, the results suggest the usefulness of the combination of texture and color features for automated VSS-based scar rating. However, the technical analysis for the best texture-color feature combination has been an open issue^35^, and needs further study. Another issue is about the class imbalance in pattern classification^32^, in which some classes are represented with larger training samples while others with only a few. Here, each of the classes for the VSS total scores of 4, 5 and 8 has only 9 samples, while the classes for the VSS total scores of 1 and 7 have 81, and 45 samples, respectively. However, for ECOC-OSVM, the number of misclassification for the VSS total scores of 4 and 8 is only one while that for the VSS total scores of 5 is zero, suggesting that the class imbalance is not a hindrance to the present classification. Similar observations are also found in the classification using ECOC-SVM and ECOC-*k*-NN.

It has been reported that various objective measures for burn scar assessment can be based on color, texture, dimensions, biomechanical properties, pathophysiological disturbances, tissue microstructure, and pain/sensation^7^. Here, although based on the VSS evaluation for rating the total scores of the scars, we only take into account the information of color and texture, where the first relates to erythema and pigmentation that significantly reveal the appearance of a scar, and the latter is concerned with the smoothness, roughness, and irregular characteristics of a scar surface that has a significant effect on the subjective opinion about the scar^7^. Pliability and height criteria of the VSS were not included in the feature extraction process. Taking into account 3-dimensional analysis is immediately feasible in the proposed method to include thickness and volume in the classification so that the computerized assessment can be more robust, while other properties and factors of burn scars can be independently augmented to further enhance the assessment.

An objective burn scar assessment using finite element analysis of salient image features and elastic property of scars was carried out in^15^ with the inclusion of four patients. However, this study only suggested the correlation between subjective VSS-based rating with the relative elastic indices that is inconclusive due to the limited data. In general, with further exploration of other methods for extracting effective texture, color, and dimensional features of the scar together with advanced machine learning methods and sufficient training data, we expect that the proposed approach for automated assessment of burn scars would be clinically useful in terms of the trade-off of costly objective scar measuring devices.

The *k*-fold cross-validation is a method to improve over the holdout validation, where the data set is separated into a training set and a testing set. For the *k*-fold cross-validation, the data set is divided into *k* subsets, and the holdout method is repeated *k* times. For each validation, one of the *k* subsets is used as the test set and the other *k*-1 subsets are put together as a training set. The *k*-fold error is computed as the average error for all *k* trials. The leave-one-out (LOO) cross validation is the *k*-fold cross validation, with *k* equal to *N* that is the number of data points in the set. This indicates that the classifier is trained for *N* times with the data except for one sample, and a prediction is made for the untrained sample. In this study, different inter-appearance scars (sub-image samples obtained by dividing different scars) and different intra-appearance scars (sub-image samples obtained by dividing the same scar) were used to test the algorithm, where information about the pliability and height of the scars were not possibly taken into consideration by means of two-dimensional data in the training and testing phases. The classification with the exclusion of the pliability and height of the scars resulted in 85% accuracy. It can be expected that if the two sources of information were included in the classification, the cross-validation rate could have been higher or possibly as high as 98% accuracy that was obtained with the two-score tolerance as shown in Table 4.

## Conclusion

Texture-color image analysis and machine learning methods for automated evaluation of burn scars using training data labeled with the VSS total scores have been presented and discussed. The cross-validation accuracies obtained from the error-correcting output coding with an optimized support vector machine learner using combined texture and color features are promising for overall scar rating as an objective and reproducible measure, which is very cost-effective in comparison with the use of other objective scar measurement instruments. The proposed approach used the VSS scores for machine learning in this study, but can be used for learning other scar rating methods. Furthermore, texture and color features of the phenotypic characteristics of burn scars can be useful for gaining insight into the association between the genetic information and wound outcomes of patients^36^. The proposed approach is also potential for the development of telemedicine-based assessment of burn scars via the transmission of digital images from cell-phone based multimedia^37,38^.

In this study, only Caucasian pediatric patients and VSS total scores being up to 9 were included in the VSS-based automated assessment due to the data availability. Automated VSS-based assessment of non-Caucasian patients, including children and adults as well as the full range of VSS total scores, will be our on-going and future investigation when more clinical data become available. The automated classification would be more effective if different automated scar assessment systems will be designed according to skin colors of different races, age groups, and gender.

In summary, we have developed an automated assessment approach that can reliably produce VSS scores to match those provided by human medical experts. Using other scoring systems such as the POSAS or other objective measurement tools for studying the correlation with the VSS to be incorporated in automated image analysis is worth considering in our future research.

### Data and software availability

Image data and Matlab codes used in this study are available at the first author’s (T.D.P.) personal homepage: https://sites.google.com/site/professortuanpham/codes.
